# Reengineering a thermostable keratinase past the boiling point

**DOI:** 10.1128/aem.00126-26

**Published:** 2026-06-02

**Authors:** Francis E. Jenney

**Affiliations:** 1 Biomedical Sciences, Philadelphia College of Osteopathic Medicine, Suwanee, Georgia, USA; Michigan State University, East Lansing, Michigan, USA

**Keywords:** keratinase, *Thermoactinomyces*, protein computational design, rational design

## Abstract

Millions of tons of keratin-rich waste are disposed of annually, which could be revalorized, mitigating greenhouse emissions and pollution. Microbial keratinases offer tools for keratin waste valorization, yet they lack robustness for industrial use. In an *Applied and Environmental Microbiology* article, Y. Yang, Y. Luo, Y. Ding, Y. Yao, et al. (92:e01860-25, 2025, https://doi.org/10.1128/aem.01860-25) integrated machine learning, rational design, and empirical screening to produce boiling-resistant variants that were reusable and highly effective at keratin degradation, demonstrating the promise of deep-learning-guided enzyme engineering.

## COMMENTARY

The circular economy shifts the linear “harvest → use → discard” model to a sustainable system that reuses, rather than disposes of, and even revalorizes wastes (1). This principle applies broadly to many industrial waste products, from plastics (2) to waste food (3) and even water (4). Microbial recycling and reprocessing will undoubtedly be a major force in this effort (2). As of 2024, the United Nations Environment Programme estimated that worldwide >2 billion tons of municipal solid waste are produced annually, of which less than 20% is recycled (5). Astonishingly, a bit more than half of this is food waste (3), and while food waste attributed to meat may be as low as 4% of the total on average, meat production has a higher economic cost. Aside from animal products used for food, a major aspect of animal husbandry is the production of fibrous, keratin-rich materials, such as hair, fur, hides, hooves, claws, scales, and feathers, and there is also significant production of human hair annually, virtually none of which is usable as food for humans or animals. As of 2022, it was estimated that more than 16 million metric tons of keratin-rich materials are produced per year (approximately 1/3 of which is feathers), which could potentially be used, for example, to replace some of the >70 million tons of petroleum-based fibers used each year (6). Some of this waste can be and has been converted to animal feed, fertilizer, or other uses at efficiencies up to 80% (6); however, the majority is simply disposed of. The millions of tons of keratinous material disposed of in landfills or incinerated generate significant amounts of pollutants, such as methane, CO_2_, NOx, and SOx, since most of the mass is protein (7). Valorization of keratin-containing animal waste is both an economic opportunity and an environmental necessity, and prokaryotic enzymes are a vital source of tools to facilitate this aim. In an *Applied and Environmental Microbiology* article (8), Y. Yang et al. combine a number of rational and empirical methods to optimize the stability and function of a keratinase enzyme from a thermophilic bacterium for “polyextreme” industrial conditions.

Keratins, a diverse family of fibrous, water-insoluble proteins, form the major structural mass of epithelial cells and of various protective structures in mammals, birds, and reptiles, including hair, fur, and feathers, reviewed in reference 9. Despite being rarely mineralized like bone (except in, e.g., whale baleen), animals can make highly flexible and extremely strong materials with specialized cells (keratinocytes) containing as much as 85% of keratin per cell mass. Keratins are sometimes grouped as acidic (type I) and basic (type II); alternately, they can be grouped as soft (with ~1% sulfur content, found mainly in skin cells) and hard (with ~5% sulfur, found in hair and nails) (9). They form intermediate filaments in many cells and are divided generally into four major types: alpha, beta, feather, and amorphous, based on structure. Most organisms have dozens of genes encoding keratins, with many different types of heterogeneous fibers formed (7). The compact and various nature of the keratin filaments, their insolubility in water, and the mechanical strength derived from (among other bonds) many intra- and interchain disulfide bonds make them difficult for proteases to access (10). However, the protein content (as high as 95% keratin in some wools [7]) makes them a valuable resource, the majority of which is currently wasted. Given this high protein content, it behooves us to find methods to reuse the megatons of keratin-rich materials discarded annually and decomposing in landfills, creating pollution and wasting immense resources (7).

This decomposition process is pursued naturally by prokaryotes and fungi, and their protease enzymes have been extensively studied to elucidate mechanisms and ultimately engineer improvements (11). Enzymes from extremophiles have been studied for decades for use in industrial and food applications, as their enzymes tend to be both robustly stable under the desired conditions (cold or heat) and highly active. Proteases can be broadly grouped by their mechanism of action (metalloproteases, cysteine, serine, or aspartate) and/or their optimal pH (acidic, basic, or neutral), as well as whether they function as exo- or endopeptidases (12). Keratin is quite resistant to most proteases and is highly stable, as shown, for example, by successful DNA isolation from intact bighorn sheep hairs 9,800 years old (13). Nonetheless, keratinous materials do eventually decay, and this is primarily catalyzed by various keratinases. Enzymes referred to as keratinases are not specific for keratin but typically fall under the serine protease family of subtilisins (IUBMB Enzyme Nomenclature EC 3.4.21.62 or MEROPS database designation S8) (14). No single enzyme can completely degrade all keratin-rich material, but consortia of bacteria, actinomyces, and fungi will complete the process (15). Many fungi have shown their ability to degrade keratin and can be found both as animal pathogens and saprophytes. The ubiquitous laboratory reagent Proteinase K, derived from the dermatophyte fungus *Tritirachium album*, which is used to remove protein from nucleic acid samples, can degrade keratin. The non-pathogenic saprophytic fungus *Onygena corvina* secretes a complex cocktail of proteases and other enzymes, which can degrade keratin, and *Aspergillus*, *Trichoderma*, and many other fungi have demonstrated keratinase activities (15).

Many prokaryotic species have been analyzed in search of useful tools for keratin digestion. There are Gram-negative aerobic strains of *Pseudomonas aeruginosa* and *Alcaligenes faecalis* which can efficiently hydrolyze chicken feathers, as well as Gram-positive bacteria such as *Microbacterium* (16). Many species in the *Bacillus* genus (17) produce keratinases, and some from *B. licheniformis* are used commercially (15). Another type of Gram-positive filamentous soil bacteria, the Actinomycetes contain many members; for example, *Streptomyces* species can digest keratin (18). Subtilisin-like serine proteases from extremophiles, in particular hyperthermophiles, which can be resistant to thermal unfolding and detergents, are being tested to degrade recalcitrant prion proteins (19) or keratin like *Geoglobus acetovorans* (20). Keratinases from thermophiles have also been sought which can function at higher temperatures to increase the speed and efficiency of digestion, for example, from *Fervidobacterium* strains (in the phylum Thermotogota) and *Thermoanaerobacter* (15).

The experiments conducted by Yang et al. (8) target the keratinase of *Thermoactinomyces vulgaris* CDF to extend its ability to digest feathers. The *Thermoactinomyces* are a type of Gram-positive, filamentous, spore-forming soil bacteria related to *Bacillus* (21). *T. vulgaris* (Tv) is the type species isolated in 1899 from a decaying straw/manure mixture, and it secretes a keratinase, a subtilisin-type protease referred to as C2, which has been demonstrated to degrade intact chicken feathers (22). Keratinase C2 is well characterized *in vitro*, demonstrating Ca^2+^ dependence and optimal activity at pH 10 and 85°C, and it is resistant to some stresses such as salinity and detergents (22). However, C2 degrades at higher temperatures, and processing of large amounts of keratinous materials at boiling temperatures would be preferred, as this would allow sterilization of the essentially always contaminated raw materials and increase the speed and efficiency of digestion (22). The authors generated a highly functional, boiling-resistant version of C2 using empirical engineering design combined with rational design, machine learning predictions, and molecular dynamics calculations. In addition to successfully engineering a useful protein, they have generated important data that can be used in training structure prediction algorithms.

Proteins have evolved under many constraints both biophysical (pH, stability, and interaction with partners) and functional (structure and catalysis) (23), and may have characteristics not specifically necessary for their function; for example, protease C2 functions *in vitro* optimally at pH 10 and 85°C, while *T. vulgaris in vitro* grows optimally at 50–55°C and pH ~7 (21). Native enzyme stability and functionality have not been optimized for human industrial use, as extremes of temperature and pH are often preferred, and since the tools became available—first random mutagenesis, then directed evolution techniques have been tested—to increase solubility, stability, and enzyme activity (24). Information from alignment of homologous amino acid sequences, comparing older branching thermophilic sequences and structural factors of homologs, has been incorporated, and “semi”-rational design, along with these computation tools, have been used to some success. More recent machine learning tools, such as AlphaFold and RoseTTAFold (reviewed in reference 25), have shown great power in predicting protein structures. Yang et al. utilized five automated computational packages to predict mutations in *T. vulgaris* protease C2 that might increase thermostability. There was significant variation in the prediction results (from 7 to 119 proposed changes in different models). Interestingly, only a single amino acid change (serine at position 182 to alanine) was predicted by all five, although it did not turn out to be useful. They chose a total of 35 mutants, nine predicted by a single software and 26 mutants predicted by at least two programs, expressed these in *Escherichia coli*, purified the recombinant proteins, and tested residual activity after heat treatment at 95°C. Only four mutants showed a significant increase in thermostability, and only one, Ser130Asn, was predicted by four of the five models. Another variant containing seven mutations at multiple residues precipitated after heating.

Many subtilisins from hyperthermophilic organisms have large numbers of acidic surface residues, some of which are involved in forming ion pairs or binding the Ca^2+^ ion cofactors, thereby increasing thermostability. Thus, the authors applied structure-based rational design to modify surface ion pairs which could interact with the Ca^2+^ cofactors. Of the eight mutations tested, only one increased thermostability. Testing variants which modified either the number of acidic surface residues or surface ion pairs, only one position—modifying a polar asparagine residue at position 94 to an acidic residue (aspartate or glutamate, N94D/E)—showed improved stability, and another variant, modifying a polar glutamine at position 20 to an acidic aspartate residue (Q20E), did not form an ion pair as predicted *in silico* but did contribute to increased thermostability. Another technique demonstrated to increase protein stability is the introduction of proline residues in protein secondary structure β-turns. The rigid side chains of proline typically cause abrupt shifts in the direction of the amino acid chain but also tend to stabilize these turns. In protease C2, only two of the nine positions tested with proline substitutions increased stability. One surface substitution (Q20E), which showed significantly increased stability, and three surface substitutions increasing the negative charge on the surface of the protein (N63D, Q123D, and S175D), none of which had a significant effect on stability, were combined to test for synergistic effects. The resulting mutant C2 protease construct, SM4, containing all four mutations, showed an increase in stability after 1 h at 90°C. Five more stabilizing mutations tested after computational prediction were added to SM4 to create variant SM9, which showed a remarkably higher thermostability and >97% residual activity after 1 h at 90°C.

Protein structures are typically viewed as static snapshots from X-ray crystal structures; however, proteins are dynamic molecules, constantly in motion. This motion can be simulated *in silico* by molecular dynamics (MD) up to the microsecond timescale, enabling useful predictions of the role of amino acid residues in structure and function. Comparisons of sequences between a number of proteases from thermophiles allowed the authors to identify a number of potential “hot spots,” where thermostabilizing elements such as disulfide bonds and Ca^2+^-binding sites are increasing thermostability. Data from MD simulations were consistent with some of these regions in the thermostable SM9 mutant of C2 becoming more rigid, increasing the thermostability relative to the native C2 enzyme.

Subtilisins all have at least one Ca^2+^ cofactor binding site (26); thus, several experiments were conducted to modify the two existing Ca^2+^ binding sites in C2, as well as to add several others found in homologous proteins. Modifying the existing calcium binding sites led to a decrease in stability, but addition of new Ca^2+^ binding sites found in homologs led to a net increase in thermostability in variant CM1 in the presence of added CaCl_2_. Through several cycles of modifications, the surface mutations from variant SM9 were added to CM1 to create the final product, CM16, containing a total of 16 mutations. The native C2 protein is essentially completely inactive after just 15 min at 95°C; however, the three mutants produced here by a combination of computational and rational design—CM1, SM9, and CM16—had respective half-lives of 1.5, 8, and 50 h at 95°C, the last representing a 200-fold increase in stability. It is important to note that virtually all the mutants generated retained about the same activity at 60°C as the native C2 protein, indicating that these mutations primarily affected thermostability, not activity at the native growth temperature. The SM9 and CM16 variants were further tested for their ability to withstand heating at 110°C, retaining 58.1% and 94.6% residual activity, respectively, which is comparable to pyrolysin (82.3%), from the hyperthermophile *Pyrococcus furiosus*, although this is dependent on the type of buffer used. Not only was stability enhanced, but protease activity was enhanced at higher temperatures, with the optimal temperature for SM9 and CM16 activity now being 100°C.

These remarkable results demonstrate that proteins can be modified to dramatically increase stability while retaining or enhancing enzymatic activity. Further testing of other stressors, such as urea, organic solvents, and detergent (sodium dodecyl sulfate), showed that some of the variants had significantly increased resistance to the stress and, in the case of organic solvents, dramatically increased resistance. The SM9 and CM16 variants were then tested side by side with the native C2 protein and proteinase K as a control for the ability to degrade intact feathers with and without 10 mM CaCl_2_. Assessed visually, SM9 and CM16 completely degraded feathers within 4 h at 95°C, while the control proteins suffered thermal degradation. The degradation products appeared to precipitate in the presence of 10 mM CaCl_2_, as assessed by the release of soluble peptides, so reducing the calcium concentration to 1 mM and the temperature to 70°C allowed complete disintegration of the feathers. Their final result is a protocol to cycle from 95°C with high calcium to 70°C with low calcium with variant SM9, which allowed recycling of the stable enzyme multiple times to completely degrade native chicken feathers. Interestingly, they inadvertently created a “kill switch” for the enzyme; by adjusting the Ca^2+^ concentration or removing it with chelators, protease activity can be removed or tuned when desired, since the stability of the enzyme is calcium dependent.

The paper by Yang et al. is the first report of a subtilisin being modified to be resistant to boiling. The combination of structure-predicting algorithms, rational design comparing other thermostable subtilisins, and empirical testing has created a new potential tool to degrade feather waste into soluble peptides. Other groups are addressing similar problems with disposal of different insoluble natural polymers, for example, chitin in the tens of millions of tons of mollusk and crustacean shell waste discarded every year (27), which would profit from the development of similar molecular tools. Perhaps more importantly, this study provides valuable experimental data which may improve the five machine learning systems used in this work, as well as other systems in development. While these systems have great predictive power (the AlphaFold team was awarded a Nobel Prize for their advancements in *ab initio* protein structure prediction), much work remains (24). As demonstrated by Yang et al., many mutations predicted by software had little effect on thermostability, consistent with many studies showing that the majority of mutations are destabilizing or have, at best, minimal effects on structure. In the present study, though some mutations did combine synergistically to increase stability, this could only be determined (so far) by empirical testing. Experimental validation of predicted structures and functions is vital for training and optimizing deep learning networks (28), which should allow rapid development of molecular tools derived from nature that can be tailored for industrial use. In terms of thermostability considerations, the vast majority of structures available in the Protein Data Bank (as of April 2026 >250,000 structures and ~1 million computed structure models; https://www.rcsb.org/stats/) were determined at cryogenic temperatures. Only relatively recently have structures been determined closer to physiological conditions, at or above room temperatures (29) or extreme native temperatures (30), demonstrating changes or flexibility in structures not observed in cryogenically analyzed crystals. Adding experimental data to these prediction engines is critical to reaching the goal of precision engineering of enzymes to deal with the many biological and chemical wastes produced by human civilization, and the report by Yang et al. is an important addition to this data set.
